# Corneal permeability changes in dry eye disease: an observational study

**DOI:** 10.1186/s12886-016-0231-3

**Published:** 2016-05-13

**Authors:** Kenji Fujitani, Neha Gadaria, Kyu-In Lee, Brendan Barry, Penny Asbell

**Affiliations:** Department of Ophthalmology, Icahn School of Medicine at Mount Sinai, New York, NY 10029 USA

**Keywords:** Fluorophotometry, Dry eye, Keratoconjunctivitis sicca, Ocular surface disease, Corneal permeability

## Abstract

**Background:**

Diagnostic tests for dry eye disease (DED), including ocular surface disease index (OSDI), tear breakup time (TBUT), corneal fluorescein staining, and lissamine staining, have great deal of variability. We investigated whether fluorophotometry correlated with previously established DED diagnostic tests and whether it could serve as a novel objective metric to evaluate DED.

**Methods:**

Dry eye patients who have had established signs or symptoms for at least 6 months were included in this observational study. Normal subjects with no symptoms of dry eyes served as controls. Each eye had a baseline fluorescein scan prior to any fluorescein dye. Fluorescein dye was then placed into both eyes, rinsed with saline solution, and scanned at 5, 10, 15, and 30 min. Patients were administered the following diagnostic tests to correlate with fluorophotometry: OSDI, TBUT, fluorescein, and lissamine. Standard protocols were used. *P* < 0.05 was considered significant.

**Results:**

Fifty eyes from 25 patients (DED = 22 eyes, 11 patients; Normal = 28 eyes, 14 patients) were included. Baseline scans of the dry eye and control groups did not show any statistical difference (*p* = 0.84). Fluorescein concentration of DED and normal patients showed statistical significance at all time intervals (*p* < 10^−5^, 0.001, 0.002, 0.049 for 5, 10, 15, & 30 min respectively). Fluorophotometry values converged towards baseline as time elapsed, but both groups were still statistically different at 30 min (*p* < 0.01). We used four fluorophotometry scoring methods and correlated them with OSDI, TBUT, fluorescein, and lissamine along with adjusted and aggregate scores. The four scoring schemes did not show any significant correlations with the other tests, except for correlations seen with lissamine and 10 (*p* = 0.045, 0.034) and 15 min (*p* = 0.013, 0.012), and with aggregate scores and 15 min (*p* = 0.042, 0.017).

**Conclusions:**

Fluorophotometry generally did not correlate with any other DED tests, even though it showed capability of differentiating between DED and normal eyes up to 30 min after fluorescein dye instillation. There may be an aspect of DED that is missed in the current regimen of DED tests and only captured with fluorophotometry. Adding fluorophotometry may be useful in screening, diagnosing, and monitoring patients with DED.

## Background

Keratoconjunctivitis sicca or dry eye disease (DED) is one of the most common ocular conditions that patients seek care for and affects as many as 11.4 % of men and 16.7 % of women [1]. Current treatment options range from artificial tears to anti-inflammatory and immunosuppressant agents, and treatment has shown to drastically improve quality of life and prevent damage to the ocular surface [2]. The most common methods to diagnose DED include a comprehensive eye exam, symptom survey with Ocular Surface Disease Index (OSDI), tear breakup time (TBUT), lissamine green staining (lissamine), Schirmer’s, and corneal fluorescein staining (fluorescein), but these diagnostic tests have a great deal of variability and potential for bias, making objective and accurate DED diagnosis and management difficult [3].

Miyata et al. suggested that a fluorophotometer could measure corneal epithelial permeability due to the correlation between fluorescein concentrations in the cornea and corneal damage [4]. Specifically, fluorophotometry could evaluate ocular surface epithelium barrier function [5]. Although some degree of corneal staining is inevitable and found on 79 % of corneas, the majority of the dye should remain in the tear film on the surface of the eye and not adhere to the eyes as the corneal epithelium protects the underlying layers of the cornea under normal conditions [6, 7].

Using the Fluorotron Master fluorophotometer (Ocumetrics, Mountain View, CA), we showed in our previous study that differentiating between DED and normal patients depending on the fluorescein concentration in the cornea at certain time intervals was possible [8]. This study aims to test the capability of the fluorophotometer further: we investigated whether fluorophotometry correlated with already established diagnostic tests for dry eye disease and whether it could serve as a minimally invasive objective metric to evaluate dry eye disease.

## Methods

After receiving approval from Icahn School of Medicine at Mount Sinai’s Institutional Review Board, prospective patients were recruited for this observational study. Informed consent was obtained from all subjects. The study was carried out with patients who visited the Faculty Practice of Department of Ophthalmology at Icahn School of Medicine at Mount Sinai in New York, NY.

Data were collected from normal and dry eye patients, who were followed for DED by an ophthalmologist prior to the study for 6 months or more. Patients were classified as normal or DED according to their clinical diagnosis. DED patients must have had at least 2 signs and/or symptoms of dry eyes such as foreign body sensation, ocular irritation, light sensitivity, burning and grittiness, itchiness, edema of lid, conjunctiva and cornea, or hyperemia in order to be included in the study. Normal subjects who had no signs or symptoms of dry eyes or other ocular problems except for refractive error served as controls. Patients with any conditions that limited their ability to understand the consent were excluded from the study. Pregnant patients, those under 18 years of age, patients with acute or sub-acute inflammation/infection of the anterior segment of the eye, and those with history of allergy to fluorescein were also excluded from the study.

All fluorophotometry of the cornea was performed with the Fluorotron Master flurophotometer (Ocumetrics, Mountain View, CA) in accordance to the manufacturer’s instructions and the provided scanning software. Each eye had a baseline fluorescein scan performed prior to any introduction of fluorescein dye to measure each eye’s intrinsic fluorescence. 50 μl of 1 % sodium fluorescein dye was then placed into both eyes. Two minutes later, the fluorescein was thoroughly rinsed with 100 μl of non-preserved normal balanced saline solution. Fluorescein scans were started immediately after washing and recorded at 5, 10, 15, and 30 min thereafter, beginning with the OD eye. The fluorescein scan involved no direct contact between the eye and the device. The corneal peak values of fluorescein concentration were recorded. The following diagnostic tests were also administered to patients with DED to correlate with fluorophotometry results: OSDI, TBUT, fluorescein, and lissamine. Standard protocols were used for the DED diagnostic exams. TBUT was observed under cobalt blue illumination, once per eye. We used the National Eye Institute scale for grading fluorescein corneal staining and the Van Bijsterveld scale for grading lissamine green staining of the conjunctiva.

Corneal fluorescein levels at multiple time points in both eyes were recorded into Microsoft Excel (Microsoft, Redmond, Washington). Spearman’s correlations were used to detect any correlations between fluorophotometry and other DED diagnostic tests. Microsoft Excel and SPSS version 20.0 (SPSS Inc., Chicago, IL) were used for statistical analysis. *P* < 0.05 (two-tailed) was considered significant.

## Results

**Fig. 1:**
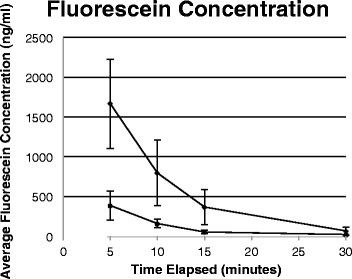
Average fluorescein concentration in dry eye patients. Legend: Fig. 1 depicts the increase in corneal peak fluorescein concentrations (ng/ml) from baseline at 5, 10, 15, and 30 min time intervals for dry eye disease and normal patients. The top line with diamond markers represents dry eye disease patients, and the bottom line with square markers represents normal patients. Error bars represent the 95 % confidence interval. Fluorescein concentrations converged towards its baseline as time elapsed, but were still statistically different from its baseline at 30 min (*p* < 0.01)

We included 50 eyes from 25 patients (DED = 22 eyes, 11 patients; Normal = 28 eyes, 14 patients). Baseline scans of the dry eye (22.19 ± 8.05 ng/ml) and control (21.81 ± 5.17 ng/ml) did not show any statistical difference (*p* = 0.84). Figure 1 shows the amount of increase in fluorescein concentration relative to baseline at multiple time intervals (5, 10, 15, and 30 min). Fluorescein concentration of DED and control groups showed statistical significance at all time intervals (*p* < 10^−5^, 0.001, 0.002, 0.049 for 5, 10, 15, & 30 min, respectively). DED always had higher fluorescein concentrations than controls. Fluorescein concentrations in both groups converged towards their baseline as time elapsed, but were still statistically different from their baseline at 30 min (*p* < 0.01).

Fluorophotometry values in DED group were categorized into 4 major schemes, which most clearly illustrated the change in fluorophotometry values: 1) increases in fluorescein concentration relative to its baseline at multiple time intervals (5, 10, 15, and 30 min) were labeled as ‘DIFF X’ where X represented the minutes. For instance, DIFF 10 was defined as the fluorescein concentration 10 min after washout of fluorescein dye minus the baseline fluorescein concentration of that patient. 2) log values of ‘DIFF X’ were taken and labeled as ‘LOGDIFF X.’ 3) similar to the ‘DIFF X’ scoring, percent changes in fluorescein concentration relative to its baseline at multiple time intervals (5, 10, 15, and 30 min) were labeled as ‘PERC X’ where X represented the minutes. 4) log values of ‘PERC X’ were taken and labeled as ‘LOGPERC X’.

To determine how fluorophotometry reflected the results of other DED diagnostic methods, we correlated the four fluorophotometry scoring schemes with OSDI, TBUT, fluorescein, and lissamine (Table 1). OSDI scores were adjusted into quartiles (0–25 = score of 1, 26–50 = score of 2, 51–75 = score of 3, 76–100 = score of 4) named ‘OSDI Quartiles’ to create a score that was more similar to scores of other tests. The double-digit OSDI scores were adjusted to fit with the single digit scores of other diagnostic tests. Using this, we created an aggregate score that added OSDI Quartiles, TBUT, Fluorescein, and Lissamine (Aggregate score = OSDI Quartile + TBUT + fluorescein + lissamine).

**Table 1:** Comparison of four fluorophotometry scoring schemes with other dry eye diagnostic tests

		OSDI	OSDI Quartiles	TBUT	Fluorescein	Lissamine	Aggregate score
DIFF5	Correlation Coefficient	.172	.056	.213	−.086	−.075	.030
	Significance	.444	.805	.340	.703	.739	.895
DIFF10	Correlation Coefficient	.118	−.030	.050	−.163	−.274	−.186
	Significance	.602	.895	.826	.468	.216	.408
DIFF15	Correlation Coefficient	.093	−.046	−.031	−.174	−.357	−.259
	Significance	.681	.837	.891	.439	.102	.245
DIFF30	Correlation Coefficient	.276	.256	−.118	−.037	.163	−.028
	Significance	.213	.250	.602	.870	.469	.903
LOGDIFF5	Correlation Coefficient	.172	.056	.213	−.086	−.075	.030
	Significance	.444	.805	.340	.703	.739	.895
LOGDIFF10	Correlation Coefficient	−.013	−.137	−.028	−.296	−.442*	−.364
	Significance	.955	.552	.904	.192	**.045**	.105
LOGDIFF15	Correlation Coefficient	−.040	−.156	−.120	−.307	−.534*	−.448*
	Significance	.864	.500	.605	.176	**.013**	**.042**
LOGDIFF30	Correlation Coefficient	.084	.079	−.127	−.187	−.100	−.211
	Significance	.726	.740	.592	.430	.673	.372
PERC5	Correlation Coefficient	0.000	−.105	.228	−.044	−.062	.013
	Significance	1.000	.642	.308	.845	.783	.954
PERC10	Correlation Coefficient	0.000	−.129	.071	−.107	−.296	−.188
	Significance	1.000	.568	.752	.636	.181	.402
PERC15	Correlation Coefficient	−.034	−.186	.012	−.225	−.361	−.316
	Significance	.881	.408	.958	.315	.099	.152
PERC30	Correlation Coefficient	.190	.179	−.119	.028	.252	−.003
	Significance	.396	.426	.596	.903	.258	.990
LOGPERC5	Correlation Coefficient	0.000	−.105	.228	−.044	−.062	.013
	Significance	1.000	.642	.308	.845	.783	.954
LOGPERC10	Correlation Coefficient	−.149	−.246	−.007	−.234	−.464*	−.367
	Significance	.521	.282	.975	.308	**.034**	.102
LOGPERC15	Correlation Coefficient	−.186	−.312	−.076	−.363	−.537*	−.514*
	Significance	.420	.169	.745	.105	**.012**	**.017**
LOGPERC30	Correlation Coefficient	−.019	−.017	−.114	−.096	.016	−.166
	Significance	.937	.942	.633	.688	.947	.485

Fluorophotometry values are categorized into 4 major schemes. 1) Increases in fluorescein concentration relative to its baseline at multiple time intervals (5, 10, 15, and 30 min) are labeled ‘DIFF X’ where X represents the minutes. 2) Log values of ‘DIFF X’ are labeled ‘LOGDIFF X.’ 3) Percent changes in fluorescein concentration relative to its baseline at multiple time intervals (5, 10, 15, and 30 min) are labeled ‘PERC X.’ 4) Log values of ‘PERC X’ are labeled ‘LOGPERC X.’

Fluorophotometry scoring schemes are compared with Ocular Surface Disease Index (OSDI), tear break up time (TBUT), fluorescein, and lissamine. OSDI scores were adjusted into quartiles (0–25 = score of 1, 26–50 = score of 2, 51–75 = score of 3, 76–100 = score of 4) and called ‘OSDI Quartiles.’ An aggregate score was calculated by adding OSDI quartiles, TBUT, fluorescein, and lissamine

All significance (*p*-value) are two-tailed, and *p* < 0.05 was considered statistically significant. Correlation coefficients and significance are bolded with asterisks* if less than 0.05

After correlation to other DED diagnostic tests, the four fluorophotometry scoring schemes generally did not show any significant correlations with OSDI, OSDI Quartiles, TBUT, fluorescein, and lissamine tests. Significant correlations were seen with lissamine scores and LOGDIFF 10 (*p* = 0.045), LOGDIFF 15 (*p* = 0.013), LOGPERC 10 (*p* = 0.034), and LOGPERC 15 (*p* = 0.012) and with aggregate scores and LOGDIFF 15 (*p* = 0.042) and LOGPERC 15 (*p* = 0.017).

## Discussion

DED affects a significant number of people, with prevalence ranging from 7 % in the United States to 33 % in Taiwan and Japan, but DED is difficult to diagnose and no clear gold standard to evaluate DED exists [9, 10]. Clinicians often rely on multitude of tests, including symptoms or signs and a battery of diagnostic tests, including TBUT, fluorescein, and lissamine. We strove to determine if fluorophotometry could fill that void through correlation with other established tests and better classify DED according to severity.

Past studies by Kinoshita et al. and Yokoi have found that the fluorophotometer could evaluate ocular surface epithelium barrier function using a similar methodology to our study [5, 11–13]. In one of their studies, they showed that in accordance with severity, fluorescein uptake in dry eye patients showed significant increase, and that with treatment with hyaluronan eye drops, there was significant barrier function improvement after 2 weeks [12, 13]. In our study, we evaluated not only fluorophotometry’s ability to differentiate between dry eye and normal eyes, but also the correlations of its results with different diagnostic techniques of dry eye. Among the multitude of tests available, we aimed to investigate whether fluorophotometry could outperform any diagnostic tests, combine 2–3 diagnostic tests, or become another test that measures an aspect of dry eye that is not yet captured by any of the diagnostic tests for DED.

Consistent with our previous study, this study showed that fluorophotometry could differentiate between DED and normal eyes up to 30 min post fluorescein dye instillation [8]. However, except for minor correlations with lissamine and aggregate scores at 10 and 15 min, fluorophotometry in our current study did not show any significant correlations with other DED diagnostic tests, such as OSDI, TBUT, fluorescein, and lissamine. These differences could arise because fluorophotometry encompasses a larger view of DED, taking into account the overall increased permeability of the cornea in ocular surface disease patients rather than focusing on one aspect of DED. For instance, TBUT takes into account tear production and evaporation, but fluorophotometry includes all factors that damage the corneal epithelium, and utilizes tear break up as only one component of many factors that contribute to DED.

Another possible function for fluorophotometry is to measure aspects of DED not captured by other diagnostic tests, which are somewhat imperfect themselves and thus contribute to the difficulty of objectively diagnosing DED. Dry eye patients have ocular surface disease that increase corneal permeability to fluorescein, but the amount of change as we measured it did not correlate with other tests. Fluorophotometry may include a component not yet detected with current diagnostic tests and thus could be done to rule out DED when DED is suspected, but other diagnostic tests are normal. Due to its unique position relative to other tests, adding fluorophotometry as one of its basic tests when evaluating DED may aid in diagnosing DED or in research settings studying DED. Continued research in this area could clarify this mismatch.

Fluorophotometry also did not correlate with clinical symptoms as measured by OSDI. Although there is some evidence showing OSDI can measure the severity of dry eye, the non-correlation was unsurprising since DED signs and symptoms are poorly correlated, compounded by variability between seasons, time of day, and findings among eye examinations, suggesting that symptoms may not truly reflect the whole dry eye state in patients [14, 15]. Though the cornea may be damaged, patients may still be asymptomatic. One possible explanation is that with damage to the ocular surface, the central cornea sensitivity decreases, making the patient less symptomatic [14, 16]. Similarly, the cornea may be healthy even as patients report multitude of symptoms. With fluorophotometry’s capability to differentiate dry eyes from normal eyes, one possibility could be to use fluorophotometry as one of DED screening tools to detect subclinical DED in seemingly healthy patients. Earlier detection could lead to earlier treatment, or possible prevention of progression to severe DED when it may be more difficult to intervene. Higher values of fluorophotometry in DED patients correlate with increased permeability of the cornea, so fluorophotometry could be used to measure progression of corneal damage and assess whether current treatments for DED are sufficient, adjusting as needed depending on the results [17].

This study is not without limitations. Sample size was limited, and this factor most likely contributed to the relatively high variability in fluorescein concentrations with the fluorophotometer. More eyes may reduce this variability, but the biggest likely cause of this variation is the lumping of all DED eyes into one dry eye group. Mild DED was not differentiated from severe DED, as there was no clear way to do so without a gold standard or correlations with other exams. The next step would be to explore whether fluorophotometry correlates with DED severity, especially if it may be a tool that can grade DED severity on a sliding scale. Furthermore, we cannot eliminate some of the intrinsic problems associated with fluorescein, especially at higher concentrations, and the Fluorotron Master flurophotometer itself. For instance, because the machine cannot clearly distinguish the tear film from the cornea, we utilized a similar washing method from previous studies to eliminate any excess fluorescein remaining in the tear film and to standardize the technique [8, 12, 18, 19]. Although the process was controlled as much as possible, some patients may have more or less fluorescein left on the tear film, possibly due to mechanical issues such as excessive tearing or blinking. Finally, although we aimed to capture most factors that contribute to DED, not all possible diagnostic tests were done to reduce burden on the patients. In future studies, comparing fluorophotometry to more diagnostic tests such as tear osmolarity may be beneficial.

## Conclusions

The degree of ocular surface disease as measured by fluorophotometry generally did not correlate with any other clinical DED tests. Given fluorophotometry’s capability of differentiating between DED and normal eyes, there may be an aspect of DED that is missed in the current regimen of DED tests and only captured with fluorophotometry. Adding fluorophotometry as another diagnostic test for DED may be useful in screening, assessing, and monitoring patients with DED.

### Ethics approval and consent to participate

Approval from Icahn School of Medicine at Mount Sinai’s Institutional Review Board. Informed consent was obtained from all subjects.

### Consent for publication

Not applicable.

### Availability of data and materials

If needed, data will be shared upon request.

## Abbreviations

DED: dry eye disease
DIFF: increases in fluorescein concentration relative to its baseline
LOGDIFF: log values of DIFF
LOGPERC: log values of PERC
OSDI: ocular surface disease index
PERC: percent changes in fluorescein concentration relative to its baseline
TBUT: tear breakup time
